# Metabolic activity in cryopreserved and grafted ovarian tissue using high-resolution respirometry

**DOI:** 10.1038/s41598-021-01082-z

**Published:** 2021-11-02

**Authors:** Aline Q. Rodrigues, Victor L. Picolo, Jair T. Goulart, Isabella M. G. Silva, Rayane B. Ribeiro, Beatriz A. Aguiar, Yasmin B. Ferreira, Daniela M. Oliveira, Carolina M. Lucci, Andreza F. de Bem, Fernanda Paulini

**Affiliations:** 1 Department of Physiological Sciences, Institute of Biological Sciences, University of Brasília, Brasília, DF 70910-900 Brazil; 2 Department of Genetics and Morphology, Institute of Biological Sciences, University of Brasília, Brasília, 70910-900 Brazil

**Keywords:** Energy metabolism, Infertility

## Abstract

Cryopreservation of ovarian tissue followed by transplantation represents a strategy to restore ovarian function and fertility. Stress from cryopreservation-thawing processes can lead to alterations and/or damage to mitochondrial structure and functionality. High resolution respirometry and histological analysis were used to evaluate the effect of cryopreservation and transplantation on ovarian tissue. Four different conditions were performed: Fresh non-transplanted tissue, Fresh transplanted tissue, Cryopreserved non-transplanted tissue and Cryopreserved transplanted tissue. All groups were able to respond to the substrates-uncoupler-inhibitor protocol. We found a dramatic decrease in general oxygen consumption in hemi-ovaries submitted to cryopreservation and/or transplantation. The effect of cryopreservation on mitochondrial metabolism was less intense than effect of transplantation, since the transplantation affected all of the mitochondrial states. A total of 2644 follicles were analyzed. Of these, 2198 were classified as morphologically normal. The percentage of morphologically normal follicles was significantly lower in the Cryopreserved transplanted group when compared to the Cryopreserved non-transplanted group and the Fresh transplanted group (*p*-value < 0.05). Despite decreased follicular viability and mitochondrial activity, the cryopreservation followed by transplantation of ovarian tissue proved feasible for attempts to restore ovarian function.

## Introduction

Issues related to human fertility preservation have been increasingly addressed worldwide, especially when cancer treatments are being discussed^1^. Ovarian tissue cryopreservation followed by transplantation has become a promising strategy to restore fertility^2,3^. This approach not only enables the preservation and restoration of female fertility in adult or prepubertal patients, it also facilitates the restoration of ovarian endocrine function^1,2^. Promising results have been obtained to date, with more than 200 live births^4^.

Currently, slow freezing is the most commonly used human ovarian tissue cryopreservation method^2^. However,the mitochondria and rough endoplasmic reticulum could be seriously impacted during this process^5,6^. Several studies reported mitochondrial ultrastructural damage oocytes within cryopreserved ovarian tissue^7–10^. In cryopreserved pre-antral follicles of swine^7^, caprine^8^ and bovine^10^, most mitochondria lost their cristae, while some had a granulated matrix with a number of empty spaces. According to Borges et al.^7^, the mitochondrial swelling and endoplasmic reticulum dilation could be a consequence of changes in ionic balance caused by altered plasma membrane permeability, possibly resulting from osmotic forces observed during the cryopresevation process. Although mitochondrial swelling could affect cellular metabolism, some studies showed that this process could be reversed^7,9^.

Studies report that stress arising from cryopreservation-thawing processes can lead to alterations and/or damage to mitochondrial structure and functionality, contributing, for example, to reactive oxygen substance (ROS) generation^5,11,12^. ROS can affect many cellular functions, mainly by causing oxidative damage to biomolecules (i.e. proteins, lipids and nucleic acids)^5^. Since oxidative stress determines important cellular processes, such as apoptosis, senescence and cellular signalization^5^, it is relevant to analyse the mitochondrial activity, as one strategy to measure the efficacy of cryopreservation and thawing, followed by transplantation. In this scenario, the development of new techniques to improve the efficacy of cryopreserved tissue transplantation can positively impact the process.

The study of mitochondrial physiology using isolated mitochondria or tissue biopsies can provide important data on the activity and capacity of electron transport chain (ETC), coupling between electron transportation and ATP synthesis and the oxidative stress affecting the grafted tissue. Analysis of metabolic parameters in cryopreserved/transplanted tissues, such as measuring mitochondrial oxygen consumption, can provide essential data to better understand cellular metabolism^5,11,13^.

Considering that there have only been few studies on mitochondrial activity after cryopreservation of tissues^14–16^ to date, none of which concern ovarian tissue, the aim of this study was to propose a metabolic evaluation of the criopreserved/transplated ovarian tissue to increase the inference of the quality of the samples. For that purpose, the metabolic activity in mice ovarian tissue that underwent slow-freezing cryopreservation, followed by transplantation was characterized, and matched it to histological analysis.

## Results

### Effect of cryopreservation and/or transplantation on oxygen consumption

**Figure 1:**
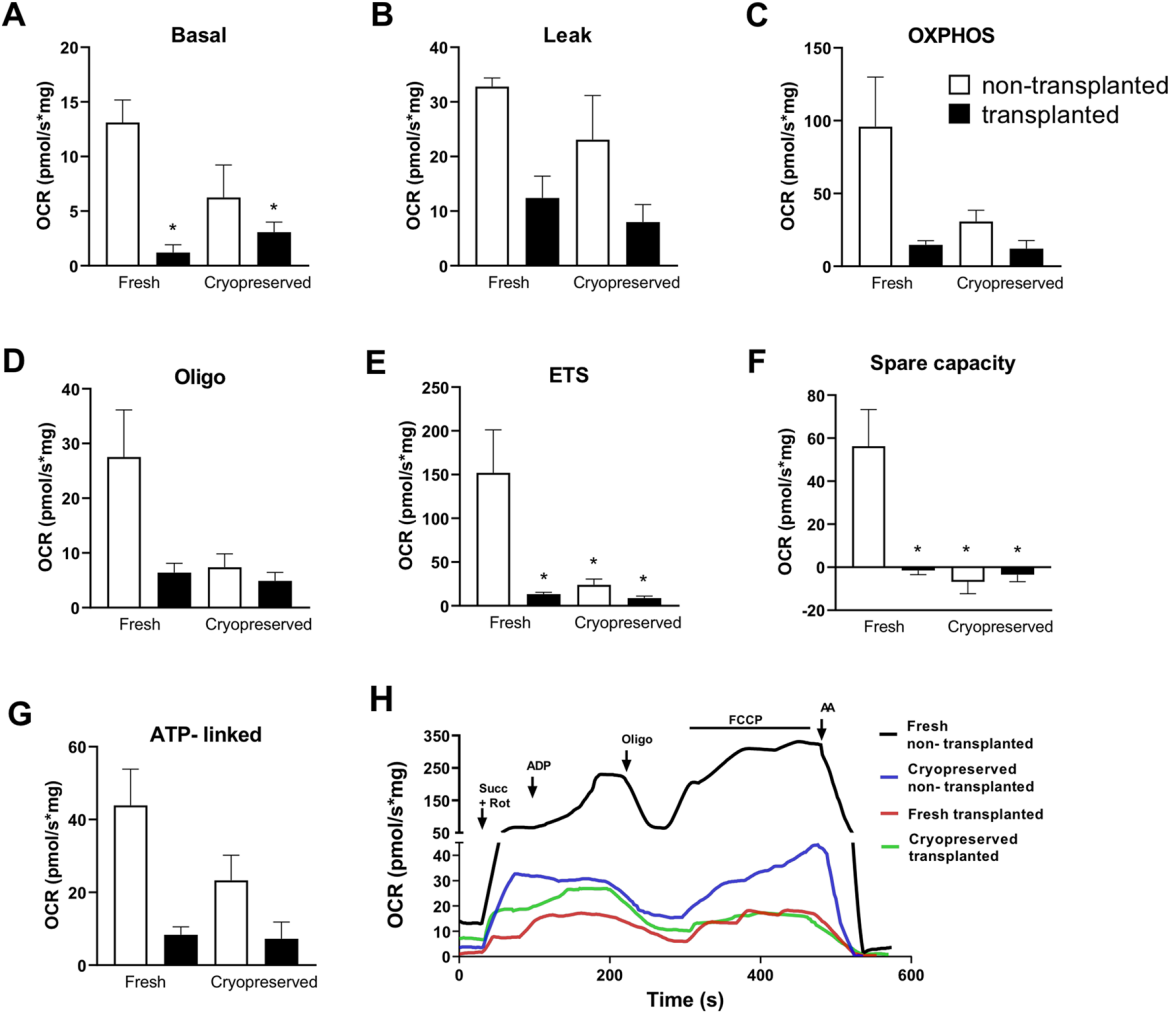
(**A**–**E**) Effect of cryopreservation and/or transplantation on oxygen consumption in homogenates of hemi-ovaries. Oxygen flux was evaluated using a substrate-uncoupler-inhibitor titration protocol (SUIT). Average O_2_ consumption rates (OCR) measured at 5 points of the evaluation, namely (**A**) Basal (O_2_ consumed due to oxidation of endogenous substrates); (**B**) LEAK (O_2_ consumed due to oxidation of exogenous succinate); (**C**) OXPHOS (OCR related to oxidative phosphorylation); (**D**) Oligo (OCR not dependent on ATP synthesis); (**E**) ETS (maximal respiratory rate resulting from uncoupled mitochondrial respiration). Assessment of reserve mitochondrial capacity (Spare capacity) (**F**) and oxygen consumption directed to ATP synthesis (ATP-linked) (**G**) were also evaluated. (**H**) Representative oxygraph trace of hemi-ovary oxygen consumption during the substrate-uncoupler-inhibitor titration protocol (SUIT) (arrowheads). Respiration rates were measured at 37 °C. O_2_ consumption was measured after sequential additions of 10 mM succinate (Succ) and 0.5 µM rotenone (Rot), 500 µM adenosine diphosphate (ADP), 0.1 µg/ml oligomycin (Oligo), Carbonyl cyanide-*p*-trifluoromethoxyphenylhydrazone (FCCP) (0.1 to 0.4 µM) and 1 µM antimycin (AA). All results were expressed as the mean oxygen consumption rates in pmol of oxygen consumed per second/mg determined by mass of tissue. Data are expressed as the mean ± SEM. *p < 0.05 compared with Fresh non-transplanted group (Two-way ANOVA followed by Tukey’s *post-hoc* test).

Adequate mitochondrial function and energy supply are vital in supporting reproductive function. Using high-resolution respirometry (HRR), we assessed the effect of the cryopreservation (fresh vs. cryopreserved) and transplantation (transplanted vs. non-transplanted) on the O_2_ consumption rates (OCR) in homogenates of hemi-ovaries. Figure 1H shows a representative oxygraph trace of a full substrate-uncoupler-inhibitor titration (SUIT) protocol of each condition. This protocol allowed us to determine the: basal OCR; oxygen consumption linked to Complex II (Leak-supported by succinate); OXPHOS (the respiratory capacity at saturating ADP concentration); Oligo (determined following the addition of oligomycin); ETS (representing the maximal OCR obtained by uncoupling the mitochondrial respiratory chain with the ionophore FCCP).

Two-way ANOVA pointed a significant effect of tranplantion proceadure in decreasing the basal OCR [F(1,15) = 14.13, *p* < 0.01] (Fig. 1A). Also, there was a significant effect for transplantation and cryopreservation interaction on Basal OCR [F(1,15) = 4.72, *p* < 0.05]. Subsequent *post-hoc* evaluation (Tukey’s test) showed that both groups submitted to the transplantation proceadure displayed a reduction in the basal oxygen consumption compared to the non-transplanted fresh tissue.

Only the transplantation significantly affected the oxygen consumption linked to Complex II [F(1,15) = 11.90, *p* < 0.01] (Fig. 1B) and to the OXPHOS state [F(1,16) = 7.86, *p* < 0.05] (Fig. 1C). Interestingly, the two-way ANOVA indicated an independent effect for the transplantation [F(1,16) = 6.49, *p* < 0.05] and the cryopreservation [F(1,16) = 5.46, *p* < 0.05] on the OCR after the inhibition of the ATP synthesis by Oligo (Fig. 1D).

Next, we evaluated the maximal OCR (ETS) through the addition of FCCP to the respiration buffer. Two-way ANOVA revealed a significant effect for both transplantation [F(1,16) = 9.68, *p* < 0.01] and cryopreservation [F(1,16) = 7.19, *p* < 0.05] on ETS (Fig. 1E). Also, there was a significant effect for transplantation and cryopreservation interaction [F(1,16) = 6.24, *p* < 0.05]. Subsequent *post-hoc* comparisons demonstrated that all treated groups displayed reduced ETS compared to the fresh non-transplanted tissue.

In addition to the respeiratory states, we calculate the OCR associated to ATP synthesis (ATP-linked = OXPHOS—Oligo) and the respiratory reserve capacity (Spare capacity = ETS—OXPHOS). The two-way ANOVA indicated that only the transplation proceadure had a significat effect on the ATP-linked OCR [F(1,16) = 7.91, *p* < 0.01] (Fig. 1G). However, both factors, transplantation [F(1,16) = 8.79, *p* < 0.01] and cryopreservation [F(1,16) = 12.57, *p* < 0.01], showed sifnificant impact on the Spare capacity (Fig. 1F). Subsequent post-hoc comparisons indicated reduced Spare capacity in all treated groups compared to the fresh non-transplanted tissue.

On collective consideration, the representative graph demonstrates an intense decrease in general oxygen consumption in hemi-ovaries submitted to cryopreservation (Cryopreserved non-transplanted) and transplantation (Fresh transplanted) when compared to the fresh tissue (Fresh non-transplanted) (Fig. 1H), possibly related to the impact caused by these treatments on the ovarian tissue. However, it is possible to observe that all groups were able to respond to the substrate-uncoupler-inhibitor titration (SUIT) protocol.

In general, the Cryopreserved non-transplanted group was more metabolically active than the others, when compared to the Fresh non-transplanted group (Fig. 1H). In fact, the impact of cryopreservation on mitochondrial metabolism was less intense than observed in transplanted hemi-ovaries. These changes can be visualized by the oxygen consumption ratios (OCR ratios) and the results found in the bidirectional ANOVA. Moreover, the Cryopreserved transplanted group had the lowest mitochondrial metabolism (Fig. 1A–G). Thus, the data suggests that the damage caused by transplantation is greater than the damage caused by cryopreservation.

Although oxygen consumption linked to the synthesis of ATP in the cryopreserved tissue (Cryopreserved non-transplanted group) was not significantly different to the fresh samples (Fresh non-transplanted group) (Fig. 1G), there is evidence that this tissue is undergoing bioenergetic dysregulation, as observed by the extinction of the mitochondrial spare respiratory capacity (Fig. 1F). On the other hand, bioenergetic dysregulation is more evident in transplanted ovarian tissue samples (Fresh transplanted and Cryopreserved transplanted Groups), due to the lower respiration associated with ATP synthesis compared with Fresh non-transplanted tissue (Fig. 1G).

### Histological analysis

A total of 2644 follicles were analyzed in all ovarian tissue samples: 831 primordial follicles, 549 primary follicles, 519 secondary follicles and 745 antral follicles, of which 2198 were classified as morphologically normal (MN). The total number of follicles, MN follicles number and the mean ± standard deviation (SD) found in each group are reported in Table 1.

**Table 1:** Number of follicles and number and percentage of morphologically normal follicles (total, primordial, primary, secondary, and antral follicles) counted in samples of fresh and cryopreserved ovarian tissue, before and after transplantation.

Groups	Total Follicles	Primordial Follicles		Primary Follicles		Secondary Follicles		Antral Follicles	
	n^o^	n^o^	n^o^ MN (%)	n^o^	n^o^ MN (%)	n^o^	n^o^ MN (%)	n^o^	n^o^ MN (%)
Fresh non-transplanted	1372 (137.2 ± 35.6)	443 (44.3 ± 19.4)	394 (88.9 ± 19)^AX^	250 (25.0 ± 8.6)	249 (99.6 ± 9)^AX^	247 (24.7 ± 20.7)	197 (79.8 ± 15)^AX^	432 (43.2 ± 15.7)	342 (79.2 ± 15)^AX^
Fresh transplanted	159 (15.9 ± 12.7)	65 (6.5 ± 6.8)	55 (84.6 ± 7)^Ax^	19 (1.9 ± 1.8)	16 (84.2 ± 2)^Bx^	71 (7.1 ± 4.4)	50 (70.4 ± 4)^Bx^	4 (0.4 ± 0.5)	3 (75.0 ± 0)^Ax^
Cryopreserved non-transplanted	985 (98.5 ± 61.6)	285 (28.5 ± 10.7)	264 (92.6 ± 10)^aX^	264 (26.4 ± 26.1)	234 (88.6 ± 24)^aZ^	170 (17.0 ± 13.7)	126 (74.1 ± 14)^aZ^	266 (26.6 ± 21)	184 (69.2 ± 15)^aX^
Cryopreserved transplanted	128 (12.8 ± 3.8)	38 (3.8 ± 1.5)	28 (73.7 ± 1)^ax^	16 (1.6 ± 1.5)	14 (87.5 ± 1.3)^ax^	31 (3.1 ± 1.7)	22 (71.0 ± 1.8)^ax^	43 (4.3 ± 3.8)	20 (46.5 ± 2.4)^bz^

n^o^ = total number of follicles, n^o^ MN = number of morphologically normal follicles. Data within parentesis are mean ± SD.

Different letters indicate significant difference (*p* < 0.01), in the same column, according to the following code:

^A,B^Difference between Fresh non-transplanted and Fresh transplanted groups.

^a,b^Difference between Cryopreserved non-transplanted and Cryopreserved transplanted groups.

^X,Z^Difference between Fresh non-transplanted and Cryopreserved non-transplanted groups.

^x,z^Difference between Fresh transplanted and Cryopreserved transplanted groups.

In general, the total number of MN follicles was lower in groups cryopreserved (Cryopreserved non-transplanted and Cryopreserved transplanted) when compared to the respective non-cryopreserved samples (Fresh non-transplanted and Fresh transplanted). Moreover, both transplanted groups (Fresh and Cryopreserved transplanted) showed a lower number of follicles compared to the respective non-transplanted groups (Fresh and Cryopreserved non-transplanted).

Although the percentage of MN primordial follicles did not differ between the groups, there is a trend in the decrease in the Cryopreserved transplanted group when compared to the Cryopreserved non-transplanted group (*p* = 0.07), as well as in the Fresh transplanted group compared to the Fresh non-transplanted groups (*p* = 0.09).

Comparing the Fresh non-transplanted group to the Fresh transplanted group, there was a reduction in the percentage of MN primary and secondary follicles after transplantation (*p* < 0.01). Moreover, comparing the Fresh non-transplanted group to the Cryopreserved non-transplanted group the same reduction was observed after the cryopreservation (*p* < 0.01). No difference was observed between Cryopreserved non-transplanted and transplanted groups, or between Fresh and Cryopreserved transplanted groups for the percentage of MN primary and secondary follicles.

Regarding antral follicles, although the Fresh transplanted group had a very low number of follicles compared to the other groups, when considering the average percentage of MN antral follicles, significant differences were found only in the cryopreserved transplanted group when compared to the cryopreserved non-transplanted and Fresh transplanted groups (*p* < 0.01).

**Figure 2:**
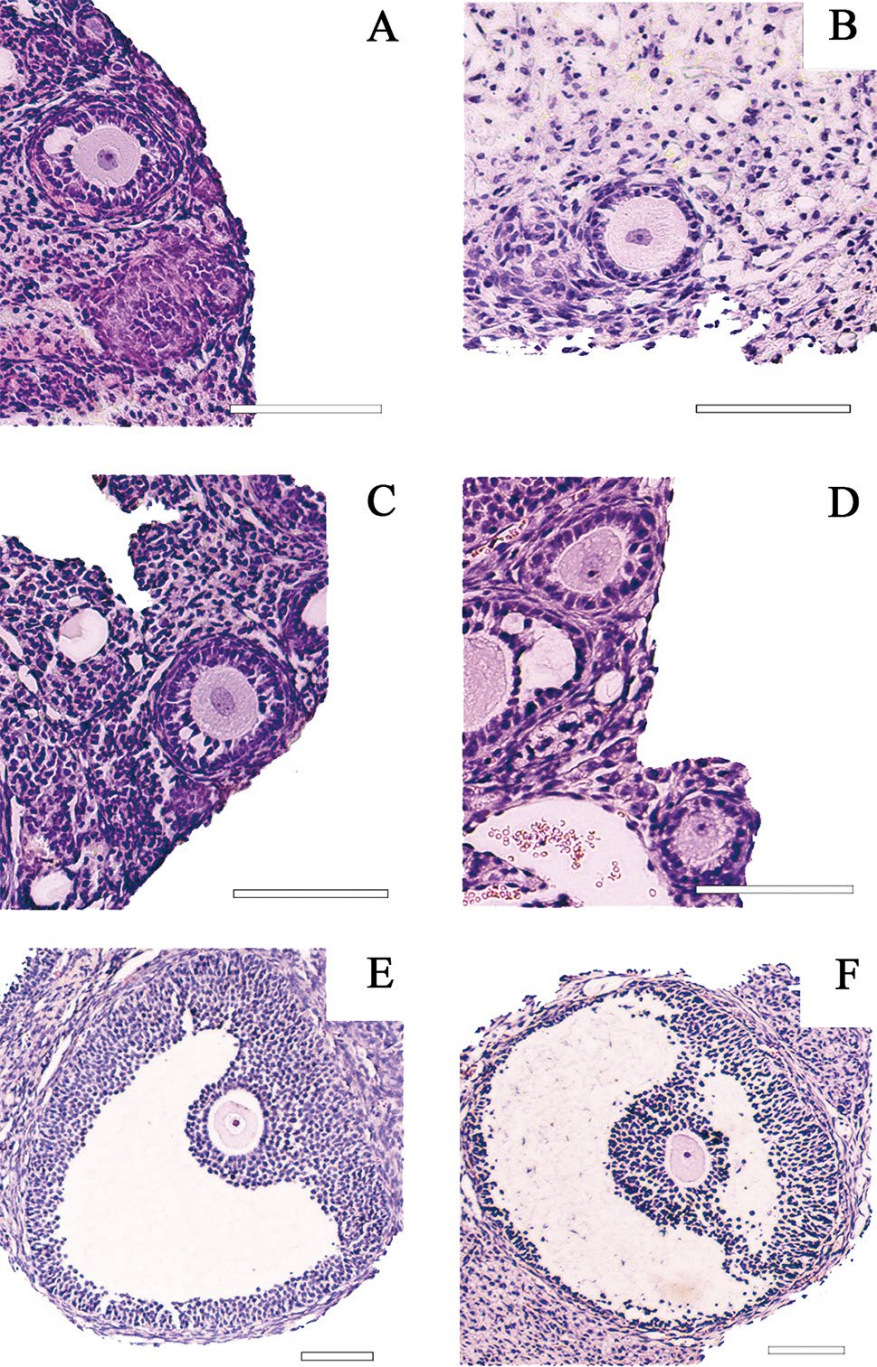
Morphologically normal follicles found in mice ovarian tissue, stained with Hematoxylin/Eosin. Fresh non-transplanted group (**A**), Fresh transplanted group (**B**,**E**), Cryopreserved non-transplanted group (**C**) and Cryopreserved transplanted group (**D**,**F**). Scale = 100 µm.

Despite the decrease in follicular viability, it was possible to find MN follicles in all analyzed groups (Fig. 2A–D). Antral follicles were also found after fresh and cryopreserved tissue transplantation (Fig. 2E–F).

Furthermore, the area of ovarian tissue occupied by stromal and follicular tissue was measured in the samples and is reported in Table 2. Fresh ovarian tissue samples (Fresh non-transplanted group) had the highest percentage of follicular tissue compared to the other groups. Thus, after cryopreservation or transplantation, the amount of follicular tissue significantly decreased (*p* < 0.05). No significant difference was observed between Fresh and Cryopreserved transplanted groups. On the other hand, the percentual area occupied by stromal tissue significantly (*p* < 0.05) increased after transplantation, both in fresh and cryopreserved tissue. No significant differences were observed between Fresh and Cryopreserved samples, both transplanted and non-transplanted.

**Table 2:** Area of ovarian tissue occupied by stromal tissue and follicular tissue (mean percentage ± SD) in samples of fresh and cryopreserved ovarian tissue, before and after transplantation.

Groups	% Follicular tissue area	% Stromal tissue area
Fresh non-transplanted	19.6 ± 11.5^AX^	75.3 ± 11.9^AX^
Fresh transplanted	3.8 ± 4.9^Bx^	86.0 ± 7.6^Bx^
Cryopreserved non-transplanted	11.9 ± 15.1^aZ^	78.8 ± 17.5^aX^
Cryopreserved transplanted	5.2 ± 9.4^bx^	87.9 ± 11.1^bx^

Different letters indicate significant difference (*p* < 0.05), in the same column, according to the following code:

^A,B^Difference between Fresh non-transplanted and Fresh transplanted groups.

^a,b^Difference between Cryopreserved non-transplanted and Cryopreserved transplanted groups.

^X,Z^Difference between Fresh non-transplanted and Cryopreserved non-transplanted groups.

^x,z^Difference between Fresh transplanted and Cryopreserved transplanted groups.

## Discussion

It is well known that ovarian tissue cryopreservation poses a major challenge, especially relating to tissue composition, with different cell types and follicles at different stages of development, which may demand different conditions for the technique. In addition, the literature shows the possible damage caused by cryopreservation and transplantation methods^1,17–19^.

Research evaluating ovarian tissue structure verified that mitochondria and rough endoplasmic reticulum cisterns (REr) are among the organelles which are more sensitive to the cryopreservation of oocyte and granulosa cells^5–9,11–14,18–20^. The aforementioned studies showed that the majority of mitochondria lost their cristae, and some presented a granulated matrix with numerous empty spaces, however, their membrane remained intact. The oximetry data showed that cryopreserved and thawed samples (Cryopreserved non-transplanted group) still possessed mitochondrial activity, albeit at a reduced level in relation to fresh tissue (Fresh non-transplanted group). According to Solaini et al.^21^, mitochondrial damage can occur as a consequence of alterations in the electron transport chain resulting from the loss of membrane potential caused by exacerbated ROS generation and/or decreased ATP synthesis. Although the Cryopreserved non-transplanted group samples demonstrated a significant decrease in Spare capacity, Oligo and ETS states compared with the Fresh non-transplanted group, it was these samples that most resembled fresh tissue in terms of metabolism.

Oximetry data of all samples confirmed that there was oxygen consumption, responsive to all reagents used, even though a significant reduction of mitochondrial function occurred. Transplanted samples, both fresh and cryopreserved, showed decreased mitochondrial function in all states analyzed (Basal; Leak; OXPHOS; Oligo; ETS; ATP-linked, and Spare capacity) compared with the Fresh non-transplanted group. These findings suggest that the damage caused by transplantation is greater than the damage caused only by cryopreservation, since it affected all analyzed mitochondrial states and the cryopreservation disturbed only three of them. This may be due to the period of ischemia and hypoxia the ovarian tissue endures immediately post-transplantation, until local neovascularization can be completely established. According to Cacciottola et al.^22^, the ischemia–reperfusion (IR) period lasts for around 2 to 5 days in mice and results in50 to 90% follicular depletion. A study by Youm et al.^23^ reported that ischemic injuries can be more harmful to tissue than cryo injuries. In accordance with this finding, our study also showed a depletion in the total number of follicles in transplanted samples, both fresh and previously cryopreserved, compared to non-transplanted samples.

It is important to note that the statistical analysis supported an interaction between the factors, that is, there was higher disturbance of mitochondrial metabolism when the tissue underwent both cryopreservation and transplantation. It is also noteworthy that ATP-linked respiration was not affected by cryopreservation, which may have contributed to better tissue viability and adaptation. Moreover, the histological analysis showed a significant decrease (*p* < 0.01) in the mean percentages of MN follicles in the Cryopreserved transplanted group compared to both the Cryopreserved and Fresh non-transplanted groups. In this sense, it was not possible to observe any significant difference between the two transplanted groups (Cryopreserved and Fresh transplanted) regarding mitochondrial activity. However, the histological analysis showed difference in fresh transplanted ovarian tissue, that showed a higher number of MN follicles in comparison to cryopreserved transplanted tissue (*p* < 0.01).

As the ovary is a complex tissue, with different structures and cell types, it is difficult to define which cells/structures within the tissue have decreased their oxygen consumption. To try to elucidate this point, we estimated the percentage of the ovarian tissue occupied by stromal tissue and follicular tissue in each treatment group (Table 2). The results of this analysis showed that after cryopreservation the tissue area occupied by follicular tissue decreased in comparison to fresh ovarian tissue samples (Fresh non-transplanted group) and decreased even more after transplantation (in both fresh and cryopreserved samples). In addition, the area occupied by stromal tissue increased after transplantation (in both fresh and cryopreserved samples), but not in cryopreserved non-transplanted samples. When looked together, the findings of this analysis and from oxygen consumption, it appears that the reduction in mitochondrial activity is more related to the amount of follicular tissue than to the stromal tissue.

Though histologic evaluation is considered the gold standard technique for ovarian reserve assessment in experimental studies, obtaining the whole picture of tissue composition and the metabolic behaviour of ovarian tissue would provide a complementary panel and improve the analysis of cryopreserved/ transplanted ovarian tissue. This issue is mainly inspired by the successful fertility preservation which has been widely achieved by ovarian tissue cryopreservation and transplant, rather than experimental, technique^24,25^.

Although it is still not possible to precisely identify which cells/structures within the tissue have decreased their oxygen consumption, the overall analysis of the tissue is an indicator of whole tissue health, which is important to sustain follicle development. It is noteworth that evaluating each part of the ovarian tissue separately would need procedures capable of generating damage to cells and follicles, compromising their methabolism. This new proposed technique for metabolic evaluation of cryopreserved and transplanted samples in association with histological analysis could indeed infer sample quality.

The transplantation of cryopreserved ovarian tissue proved to be viable in this study. Although in lower quantities, MN preantral and antral follicles were found in the tissue after freeze/thawing and transplantation for 7 days, and mitochondrial activity was observed. Other studies also evidenced ovarian tissue viability after transplantation, demonstrating the restoration of ovarian function, follicle viability and reasonable gestation rates^2,26–28^. Diverse factors still partially compromise the efficiency of cryopreservation. Reduced cell viability, increased cellular senescence and alterations of cellular functions are among the obstacles broadly associated with oxidative stress generated by this process^5^. Moreover, oxidative stress has been identified as a potential agent for some of the cryo injuries that harm cells^5^. Regarding bioenergetic activity, many cells operate at a baseline limit, requiring only part of the total capacity^29^. This way, under certain conditions, a tissue can demand a sudden increase of additional cellular energy in response to stress or increased workload, which can eventually relate to senescence or even cell death, in cases of insufficient energy resources^29^. So, after freezing–thawing the samples and restoring blood supply after periods of ischemia, mitochondrial function may be able to adapt and respond to energetic demands^11,29^.

Although the cryopreserved tissue suffered bioenergetic dysregulation, as demonstrated reductions in Oligo, ETS and Spare capacity, this tissue managed to reach an ATP-linked respiration comparable to the fresh tissue by achieving its full mitochondrial potential and depleting the Spare capacity. On the other hand, both transplanted groups also depleted the spare capacity, but did not restore ATP synthesis. Cacciotola et al.^22^ analyzed metabolic activity and oxidative stress in grafted ovarian tissue using the microdialysis technique and noted that the partial pressure of oxygen stabilizes approximately 10 days post-transplantation. Although there is no data in the literature on oxidative stress during the revascularization of transplanted tissue, it is known that reoxygenation of hypoxic tissues can lead to metabolic remodeling and mitochondrial reprogramming followed by ROS production, in addition to inflammation and cell death^30,31^. In this regard, perhaps the seven-day transplant interval analyzed in the present study was insufficient time for tissue recovery and complete restoration of mitochondrial function. Conversely, bioenergetic dysregulation was more evident in transplanted ovarian tissue samples due to the lower respiration associated with ATP synthesis. This fact may also relate to the decreased number of MN follicles in those samples because with reduced ATP availability, it is possible that the tissue reduced the energetic expenditure, leading to follicle death.

It is important to highlight that, despite bioenergetic dysregulation, the cryopreserved and transplanted ovarian tissue remained viable and may be able to regain its functionality. Future techniques aimed at protecting mitochondrial functionality may result in faster and more efficient, recovery of transplanted tissue, especially after cryopreservation. As such, antioxidant agents have been increasingly used to decrease the stress caused by cryopreservation and transplantation procedures^5,23^. Some compounds can exert effects by modulating genes responsible for cellular survival, apoptosis and oxidative stress^5^.

In conclusion, this research showed that cryopreservation of ovarian tissue has an impact on mitochondrial metabolism and follicular viability, however, it demonstrated that transplantation has a greater impact than cryopreservation per se. Transplanted samples (Fresh or Cryopreserved) showed a more pronounced decrease in the rate of oxygen consumption in different respiratory states, in addition to a lower follicle counts and lower percentages of morphologically normal follicles. Furthermore, cryopreserved tissues are even more sensitive to transplantation than fresh tissues. However, despite decreased follicular viability and mitochondrial activity, the cryopreservation technique followed by transplantation of ovarian tissue proved feasible, both morphologically and metabolically, for attempts to restore ovarian function.

## Methods

### Ethics committee and guidelines

The use of mice ovarian tissue was approved by the Ethics Committee on Animal Use of the University of Brasilia (CEUA- protocol n° 2/2018) on November 20, 2019. The study was performed in accordance with the relevant guidelines and regulations. The study was carried out in compliance with the ARRIVE guidelines.

### Animals

Twenty nude female mice *(Swiss nu/nu),* 8–12 weeks old, were used. The animals were maintained in mini-isolators in groups of five, with controlled room temperature (24 °C) and 12/12-h light–dark cycles. Mice were fed standard rodent chow and given ad libitum access to autoclaved water.

### Experimental design

The groups of animals were denominated Group 1, 2, 3 and 4. For treatment performed in ovarian tissue, the groups were named Fresh non-transplanted group, Fresh transplanted group, Cryopreserved non-transplanted group and Cryopreserved transplanted group.

The mice were randomly divided into four experimental groups (n = 5 in each group). In Group 1, animals underwent bilateral ovariohysterectomy (OSH) and the hemi-ovaries were cryopreserved. Seven days later, these hemi-ovaries were thawed and transplanted to animals from Group 2. At the same moment of receiving thawed fragments, the Group 2 animals underwent OSH during the same surgical procedure, and the hemi-ovaries recovered were immediately transplanted to Group 3 animals in. The hemi-ovaries recovered from Group 3 animals by OSH were cryopreserved and thawed 7 days after the procedure, but not transplanted. Group 4 consisted of animals euthanized immediately before the evaluation, with their hemi-ovaries considered as fresh tissue. Seven days after hemi-ovary transplantation to Groups 2 and 3, animals were euthanized, and the hemi-ovaries retrieved for analysis.

The hemi-ovary samples from all groups were divided for the analysis, with half of the hemi-ovaries fixed for histological analysis, and the other half submitted for mitochondrial oxygen consumption measurements.

**Figure 3:**
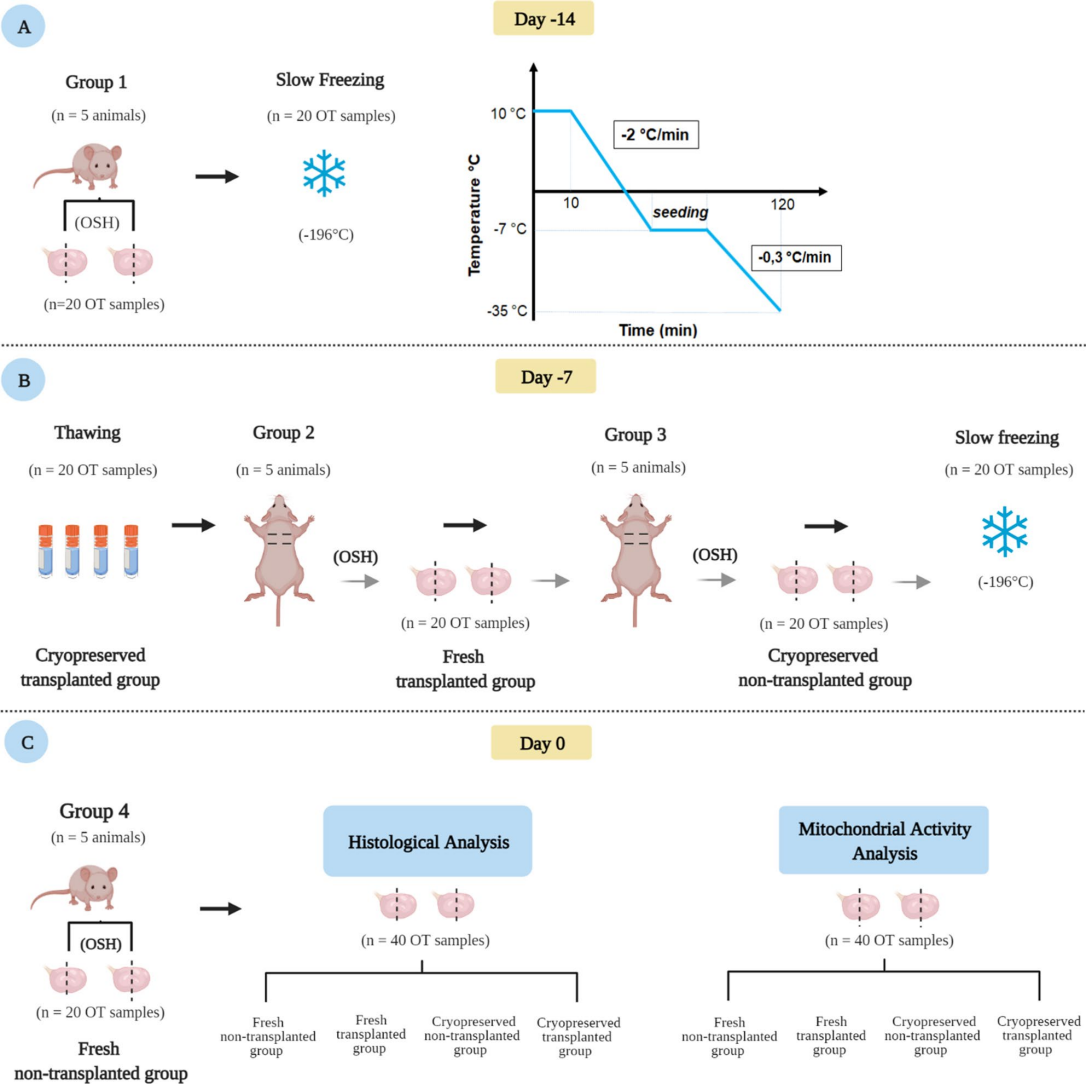
Experimental design. (**A**) Group 1 animals underwent bilateral ovariohysterectomy (OSH). Hemi-ovaries were cryopreserved and stored in liquid nitrogen (− 196 °C) for seven days. (**B**) Seven days after cryopreservation, hemi-ovaries were thawed and transplanted to Group 2 animals, forming the Cryopreserved transplanted group. Group 2 animals underwent OSH in the same surgical procedure, with the hemi-ovaries immediately transplanted to Group 3 animals, constituting the Fresh transplanted group. Hemi-ovaries from Group 3 animals were obtained by OSH, cryopreserved and thawed after 7 days, but not transplanted (Cryopreserved non-transplanted group). Group 4 animals were euthanized, and the hemi-ovaries obtained immediately evaluated as a fresh tissue (Fresh non-transplanted group). Seven days after hemi-ovary transplantation to Groups 2 and 3, animals were euthanized, and hemi-ovaries retrieved for analysis. Hemi-ovary samples from all groups, were distributed between the analysis, with half of the hemi-ovaries fixed for histological analysis, and the other used to evaluate mitochondrial oxygen consumption. OSH = ovariohysterectomy; OT = ovarian tissue, corresponding to a hemi-ovary. This figure was created by the authors using Biorender (https://biorender.com/).

Finally, samples of fresh ovarian tissue (Fresh non-transplanted group—n = 20 hemi-ovaries); frozen-thawed ovarian tissue (Cryopreserved non-transplanted group—n = 20 hemi-ovaries); fresh transplanted ovarian tissue (Fresh transplanted group—n = 20 hemi-ovaries), and frozen-thawed transplanted ovarian tissue (Cryopreserved transplanted group—n = 20 hemi-ovaries) were analyzed. The experimental procedures are schematically represented in Fig. 3.

### Surgery and euthanasia

#### Ovariohysterectomy (OSH)

All procedures were carried out under aseptic conditions. The mice were pre-anesthetized with ketamine (80 mg/kg), 10% Cetamin® and 2% xylazine (10 mg/kg, Xilazin® 2%, Syntec, São Paulo, Brazil) with anesthetic maintenance performed via inhalation of isoflurane (BioChimico, Rio de Janeiro, Brazil) vaporized in pure oxygen. Meloxicam (5 mg/kg -Maxican 2%, Ourofino, São Paulo, Brazil) and tramadol hydrochloride (12.5 mg/kg Tramal, Pfizer, São Paulo, Brazil) were administered for analgesia. The ovaries were obtained by OSH via dorsal incision and immediately dissected in half, resulting in four hemi-ovaries per animal, followed by either cryopreservation and/or transplantation, according to the experimental group.

#### Ovarian tissue transplantation

Ovarian tissue was transplanted at the time as OSH, while the animals were under anesthetic effect. Each animal had four skin incisions made in the dorsal region and one hemi-ovary inserted into each incision, into the subcutaneous tissue. All incisions were closed using non-absorbable surgical thread (Nylon 5-0) under aseptic conditions.

#### Euthanasia

At the end of the study, the animals were euthanized by anesthetic overdose followed by cervical dislocation.

### Cryopreservation and thawing

Hemi-ovaries were cryopreserved according to the changes made in the protocol described by Liu et al. (2002)^32^. Briefly, fragments were placed in cryovials containing 1 mL of slow-freezing solution (MEM, 10% SFB, 0.4% sucrose, 1.5 M DMSO), maintained at 10 °C for 10 min, then transferred to a programmable freezer (Cryogen- Neovet, Uberaba- Brazil), where they were cooled at a rate of − 2 °C/min until − 7 °C. When the temperature reached − 7 °C, crystallization was manually induced (*seeding*), and the cryovials subsequently cooled at − 0.3 °C/min to − 35 °C (Fig. 3A). Finally, the cryovials were plunged and stored in a liquid nitrogen tank (− 196 °C) for 7 days.

Prior to transplantation/analysis, cryovials were removed from cryogenic refrigeration and allowed to thaw for 1 min at room temperature/RT. Samples were subsequently transferred to a water bath at 37 °C, until completely melted. Subsequently, three successive 5-min rinses were carried out in solutions containing MEM and 10% SFB with decreasing concentrations of sucrose (0.4%; 0.2%; 0%) and DMSO (0.75 M; 0.375 M; 0 M). The hemi-ovaries were finally washed in a 1% iodine solution and 0.9% saline solution prior to transplantation. The non-transplanted hemi-ovaries submitted directly to mitochondrial and histological analysis were washed in distilled water only.

### Histological analysis

For histological analysis, hemi-ovaries were fixed in 4% paraformaldehyde for 24 h, dehydrated in ethanol, clarified in xylene and embedded in Paraplast (Sigma-Aldrich, St. Louis, MO, USA). The tissue was serially sectioned (5 μm thick), and all sections stained with hematoxylin/eosin (HE) and analyzed.

The histological sections were analyzed under a light microscope (Leica DM500, Wetzlar, Germany). Follicles were counted and classified according to developmental stage—primordial, primary, secondary and antral follicles, and tissue integrity—morphologically normal (MN) or degenerated^33,34^. Follicles that presented a uniform organization of the granulosa cells, a round oocyte and evident nucleus were classified as MN. Follicles classified as degenerated were those which presented disorganized granulosa cells, oocyte totally or partially detached from the granulosa cells, vacuoles in the cytoplasm, oocytes with a pyknotic nuclei or even follicles detached from the stromal tissue. Only structures showing an oocyte surrounded by granulosa cells were considered. Only follicles showing a visible oocyte nucleus were counted.

The area of the whole ovarian tissue area occupied by stromal tissue and follicles were obtained for each sample in three random sections (extremities and middle). Sections were scanned using EVOS M500 Imaging System (Termofisher, Brazil) and pictures were taken at ×100 magnification. The areas of the whole ovarian tissue and of all ovarian follicles and vessels (≥ 200 µm) were measured using the grid tool from ImageJ (version Fiji). Then, the percentages of the whole ovarian tissue area occupied by stromal tissue and follicles were calculated.

### Mitochondrial oxygen consumption

For tissue oxygen consumption assessment, hemi-ovaries were evaluated in a high-resolution oxygraph (OROBOROS Oxygraph-2 k Inc., Austria). First, the hemi-ovaries were weighed using a micro-analytical balance (Semi-micro scale AUW220D, Scientific Mars, Brazil). The hemi-ovaries were subsequently homogenized in 300 µL of isolation buffer solution (75 mM sucrose, 225 mM mannitol, 10 mM hepes K, 1 mM EGTA, 0.1% fatty acid-free BSA, pH 7.4), in a manual glass homogenizer. The homogenate was added to 1700 uL of respiration buffer (125 mM sucrose, 65 mM KCl, 2 mM K2HPO4, 1 mM MgCl2, 10 mM HEPES K, 0,2 mM EGTA, pH 7,2) on oroboros chamber. The mass of ovarian tissue used varied between 1.30 and 5.60 mg, therefore the O2 consumption rate (OCR) was normalized by mass of tissue (pmol O2/s/mg), measured in real time using DatLab software (Oroboros Instruments, Innsbruck, Austria).

Mitochondrial oxygen consumption, dependent on complex II in the electron transport chain (Succinate dehydrogenase), was evaluated using the substrate-uncoupler-inhibitor protocol (SUIT). Due to technical limitations in the detection of the equipment and so that the isolated effects of mitochondrial complexes were not masked, it was decided to use only complex II substrates to perform the oxygraphy.

The Basal OCR was measured without any addition of substrates, after oxygen flux stabilization. Subsequent additions of 10 mM Succinate (Succ) and 0.5 µM rotenone (Rot) were made to boost complex II and inhibit complex I, respectively; while 50 µM adenosine diphosphate (ADP; OXPHOS) and 0.1 µg/mL oligomycin (Oligo) were added to motivate and inhibit ATP synthase activity, respectively. The maximum O_2_ consumption rate was then determined by titration with an oxidative phosphorylation uncoupler: FCCP—carbonyl cyanide *p*-trifluoromethoxyphenylhydrazone [concentrations (ETS) 0.1 to 0.4 µM]. In addition to the states described, the respiration associated to ATP synthesis (ATP-linked = OXPHOS—Oligo) and respiratory reserve capacity (Spare capacity = ETS—OXPHOS) were also calculated. The experiments were performed in five repetitions, each time analyzing samples from all experimental groups to ensure the same experimental conditions for all groups.

### Statistical analysis

Statistical analysis was carried out using the two-way analysis of variance (ANOVA) with “transplantation” and “cryopreservation” as independent variables. Following significant “transplantation” x “cryopreservation” interaction, multiple comparisons were performed using Tukey’s post hoc test. Outliers were defined and then excluded after the two-sided Grubbs’ test. The Shapiro–Wilk test was used to assess normality, and the Brown-Forsythe test to assess homoscedasticity. All values are expressed as mean ± SD. Data were analyzed using the GraphPad Prism 6.0 software (GraphPad Software Inc. La Jolla, CA, USA). In the histological analysis, a comparison between the groups was made using the chi-square test, regarding the mean percentage of MN follicles. Regarding the amount of stromal and follicular tissue, the Kruskal–Wallis Test was performed. Only significant differences were documented and discussed. The differences were considered significant when *p* < 0.05.

## Data Availability

The data underlying this article will be shared on reasonable request to the corresponding author.
